# Inappropriate prescribing to the oldest old patients admitted to hospital: prevalence, most frequently used medicines, and associated factors

**DOI:** 10.1186/s12877-015-0038-8

**Published:** 2015-04-09

**Authors:** Antonio San-José, Antonia Agustí, Xavier Vidal, Francesc Formiga, Mercedes Gómez-Hernández, Juana García, Alfonso López-Soto, Nieves Ramírez-Duque, Olga H Torres, José Barbé, on behalf of Potentially Inappropriate Prescription in Older Patients in Spain (PIPOPS) Investigators’ project

**Affiliations:** Internal Medicine Service, Hospital Universitari Vall d’Hebron, Universitat Autònoma de Barcelona, Àrea General 3ª planta, Passeig Vall d’Hebron 119-129, 08035 Barcelona, Spain; Clinical Pharmacology Service, Fundació Institut Català de Farmacologia, Hospital Universitari Vall d’Hebron, Departament of Pharmacology, Therapeutics and Toxicology, Universitat Autònoma de Barcelona, Barcelona, Spain; Internal Medicine Service, Hospital Universitari de Bellvitge. Hospitalet de Llobregat, Barcelona, Spain; Internal Medicine Service, Hospital San Juan De Dios del Aljarafe, Sevilla, Spain; Internal Medicine Service, Hospital General Juan Ramón Jiménez, Huelva, Spain; Internal Medicine Service, Hospital Clínic, Barcelona, Spain; Internal Medicine Service, Hospital Universitario Virgen del Rocío, Sevilla, Spain; Internal Medicine Service, Hospital Santa Creu i Sant Pau, Universitat Autònoma de Barcelona, Barcelona, Spain; Multimorbidity and elderly patients group of the Spanish Society of Internal Medicine, Barcelona, Spain

**Keywords:** Oldest old, Polypharmacy, Potentially inappropriate medicines, Potentially prescribing omissions, Benzodiazepines, Calcium and vitamin D supplements

## Abstract

**Background:**

Scientific evidence on treatments of chronic diseases in patients 85 years old or older is very limited, as is available information on inappropriate prescription (IP) and its associated factors. The study aimed to describe medicine prescription, potentially inappropriate medicines (PIM) and potentially prescribing omissions (PPO) and their associated factors on this population.

**Methods:**

In the context of an observational, prospective and multicentric study carried out in elderly patients admitted to seven Spanish hospitals for a year, a sub-analysis of those aged 85 years and over was performed. To assess PIMs, the Beers and STOPP criteria were used, and to assess PPOs, the START and the ACOVE-3 criteria were used. To assess factors associated with IP, a multivariate logistic regression analysis was performed. Patients were selected randomly every week on consecutive days from the hospitalization lists.

**Results:**

A total of 336 patients were included in the sub-analysis with a median (Q1-Q3) age of 88 (86–90) years. The median medicines taken during the month prior to admission was 10 (7–13). Forty-seven point two per cent of patients had at least one Beers-listed PIM, 63.3% at least one STOPP-listed PIM, 53.6% at least one START-listed PPO, and 59.4% at least one ACOVE-3-listed PPO. Use of benzodiazepines in patients who are prone to falls (18.3%) and omission of calcium and vitamin D supplements in patients with osteoporosis (13.3%) were the most common PIM and PPO, respectively. The main factor associated with the Beers-listed and the STOPP-listed PIM was consumption of 10 or more medicines (OR = 5.7, 95% CI 1.8-17.9 and OR = 13.4, 95% CI 4.0-44.0, respectively). The main factors associated with the START-listed PPO was a non-community dwelling origin (OR 2.3, 95% CI 1.0-5.0), and multimorbidity (OR1.8, 95% CI 1.0-3.1).

**Conclusions:**

Prescribed medicines and PIM and PPO prevalence were high among patients 85 years and over. Benzodiazepine use in those who are prone to falls and omission of calcium and vitamin D in those with osteoporosis were the most frequent PIM and PPO, respectively. Factors associated with PIM and PPO differed with polypharmacy being the most important factor associated with PIM.

**Electronic supplementary material:**

The online version of this article (doi:10.1186/s12877-015-0038-8) contains supplementary material, which is available to authorized users.

## Background

Appropriate prescribing of medicines in the elderly and especially in the very elderly is a major clinical and economic issue. The group of the oldest old people (85 years and over) is increasing and will increase even more in western countries in the coming years. Therefore, health care and appropriate use of medicines in this group is one of the major challenges facing health care systems in these countries [1]. In this age group there is often significant multimorbidity [2,3], with limited scientific evidence available on the treatment of various chronic diseases in this aforementioned group [4]. This is due to the lack of high quality evidence on the benefits and safety of treatments for major chronic diseases in this group [5,6], and their exclusion from clinical trials [7,8]. This means that most clinical practice guidelines for major chronic diseases do not include clear recommendations for the very elderly. Therefore, to treat patients in clinical practice, an individualized approach that incorporates a comprehensive geriatric assessment is recommended [4,9-11].

Recently, medicines consumption in the oldest old has been a matter of interest [12-14]. Although to our knowledge, no studies focusing on the use and the usefulness in people 85 years old and over of the main criteria for potentially inappropriate prescription medicines, have been published.

In the context of a multicentric study focused on inappropriate prescribing of medicines in the elderly (patients 75 years old and over) in the month prior to hospital admission [6], a sub-analysis of those aged 85 years old or over was performed. The goals of this sub-analysis were: to describe the use of medicines in this very elderly group of patients, to assess inappropriate use of medicines and the associated factors, and finally to compare the results with those obtained in the group of 75 to 84 year old patients. The initial hypothesis was that, despite a shorter life expectancy and the lack of solid scientific evidence regarding the treatment of most chronic conditions in the very elderly people, polypharmacy and the percentage of potentially inappropriate use of medicines remain as high as those described for the group of 75 to 84 year old.

## Methods

An observational, prospective, multicentric study on a cohort of patients hospitalised in the Internal Medicine Services of seven Spanish hospitals was carried out for a year (from April 2011 to March 2012). The study methodology has been described in previous papers [15,16] and this is a study focusing on the oldest old patients (85 years or more).

In brief, patients 75 years or older admitted with an acute illness or an exacerbation of a chronic condition who signed the informed consent form, were included. Signed informed consent was obtained from patients or caregivers in case of cognitive impairment (dementia or delirium). Hospital admission was through either the emergency department or directly from primary care. Patients with a scheduled or a short-duration (less than 24 hours) admission, those seen as an outpatient by the researcher, and those where no access was available to primary care medical information were excluded from the study. Each hospital included 2 patients per week admitted with the inclusion criteria. Patients were selected randomly every week on consecutive days from the hospitalization lists. By design, half of the included patients were 85 years or older. Beers criteria 2003 [17], STOPP and START criteria [18,19] and ACOVE-3 underprescribing indicators for chronic conditions [16] were applied to each dataset on admission. In the study, Beers-listed Potentially Inappropriate Medicines (PIM), when at least one of the Beers criteria was prescribed, STOPP-listed PIM, when at least one STOPP criteria was prescribed, START-listed Potentially Prescribing Omissions (PPO), when at least one START criteria was omitted, and ACOVE-3-listed PPO, when at least one ACOVE-3 criteria was omitted. The study was approved by the Ethics Committee of Clinical Investigation in each participating hospital (Hospital Universitari Vall d’Hebron, Barcelona; Hospital Clínic, Barcelona; Hospital Universitari de Bellvitge, Hospitalet de Llobregat; Hospital Santa Creu i Sant Pau, Barcelona; Hospital Universitari Virgen del Rocio, Sevilla; Hospital San Juan de Dios del Aljarafe, Sevilla; Hospital General Juan Ramón Jiménez, Huelva).

Information on a patient’s characteristics and the prescribing medicines was obtained from the hospital and the primary care electronic medical records and from interviews with the patients and/or relatives, using a structured questionnaire (see online Additional file 1) [15].

Since the number of eligible patients was different for the participating centres, analyses were weighted by frequency of the eligible population in each hospital. Descriptive results for continuous and count variables are shown as median, first (Q1), and third (Q3) quartiles. Comparisons for continuous and count variables were done using regression analyses, and for categorical ones using Rao-Scott Chi-square tests. To examine the association between inappropriate prescribing and potential risk factors, a multivariate logistic regression analysis was performed where an inappropriate prescribing indicator was the dependent variable and sociodemographic variables, multimorbidity, number of prescription medicines in the preceding month before hospitalization and the other indicators of inappropriate prescription (STOPP-listed PIM, Beers-listed PIM, ACOVE-3-listed PPO and START-listed PPO) were the independent variables. The adjusted *Odds ratio* (OR) with its 95% confidence intervals (CI) was calculated. Statistical analysis was performed using the procedures for complex surveys of the SAS 9.2 program (SAS Institute Inc., Cary, NC, USA).

## Results

Three hundred thirty-six out of a total of 672 patients were included in the sub-analysis and the median (Q1-Q3) age was 88 (86–90). The main clinical characteristics of the group of the oldest old are shown in Table 1 as well as the differences between patients aged 75 to 84 years. The oldest old patients, significantly, lived more often with their families than with their partners or alone (p <0.001), had a worse functional status (p <0.0001) and a poorer cognitive baseline function (p <0.001). They were more often discharged to nursing home facilities and less often to their homes (p <0.001).

**Table 1:** **Baseline characteristics of patients (weighted percentages)**

**Baseline characteristics**	**85 and more years**	**75-84 years**	**P**
	**336 patients**	**336 patients**	
Age (median [Q1–Q3])	88 (86–90)	80 (77–82)	<0.001
Gender female (%)	60.8	53.6	0.086
Admission reason (%)			0.308
● Acute disease	54.4	50.1	
● Exacerbation of chronic disease	45.6	49.9	
Emergency room origin (%)	94.6	92.4	0.285
Dwelling (%)			0.004
● Community	82	91.5	
● Nursing Home	18	8.5	
Living with (%)			<0.001
● Partner	16.3	32.4	
● Family	51.4	38.2	
● Single	9.6	17.6	
● Others	22.7	11.8	
GP visits during previous month (%)			0.012
● None	48.1	35.9	
● One or two	41.7	51.8	
● Three or more	10.2	12.3	
Admissions during the previous month (%)			0.077
● None	85.2	84.7	
● One	14.2	12.5	
● Two or more	0.6	2.8	
Barthel Index (median [Q1–Q3])			
● Basal	60 (35–80)	80 (55–95)	<0.001
● On admission	30 (5–55)	55 (20–70)	<0.001
● On discharge	45 (15–65)	65 (35–80)	<0.001
GDS basal (%)			<0.001
● 1-2	44.1	65.1	
● 3-5	41.5	25.0	
● 6-7	14.4	9.9	
Positive CAM on admission (%)	20.38	11.66	0.004
Failures in Pfeiffer test (median [Q1–Q3])	3 (2–5)	2 (0–4)	<0.001
Charlson Index (median [Q1–Q3])	2 (1–4)	3 (1–4)	0.034
Multimorbidity (%)	67	59.3	0.060
Number of medicines (median [Q1–Q3])	10 (7–13)	10 (7–14)	0.185
Number of medicines (%)			0.400
● 0-4	9.4	6.6	
● 5-9	37.5	36.2	
● 10 and more	53.1	57.2	
Discharged to (%)			<0.001
● Home	66	80.4	
● Nursing Home	23.7	12.4	
● Another Hospital	0.7	1.2	
● Died	9.6	6	

GP: General Practitioner; GDS: Global Deterioration Scale; CAM: Confusion Assessment Method.

Median (Q1-Q3) medicines taken during the month prior to admission was 10 (7–13). The most frequently prescribed medicine was omeprazole and the main differences compared to the group of patients aged 75 to 84 were a higher prescription of acethylsalicylic acid (38.1% for those 85 years and over versus 29.7% in the group from 75 to 84 years), lorazepam (21.5% versus 15.3%), amlodipine (18.8% versus 13.2%) and paracetamol (51.5% vs. 45.2%) and a lower prescription of acenocoumarol (16.2% versus 26.0%) and simvastatin (16.4% versus 21.7%) (Table 2).

**Table 2:** **The most frequently prescribed medicines according to age groups**

**85 years old and over**		**75 to 84 years old**	
**Medicine**	**%**	**Medicine**	**%**
omeprazole	61.4	omeprazole	61.2
paracetamol	51.5	paracetamol	45.2
furosemide	47.0	furosemide	43.7
acethylsalicylic acid	38.1	acethylsalicylic acid	29.7
lorazepam	21.5	acenocoumarol	26.0
enalapril	20.8	enalapril	21.9
amlodipine*	18.8	simvastatin	21.7
metformin	18.6	metformin	21.7
nitroglycerin nitrate	17.4	ipratropium bromide	19.1
simvastatin	16.4	hydrochlorothiazide	16.9
ipratropium bromide	16.3	metamizole	16.8
acenocoumarol	16.2	lorazepam	15.3
hydrochlorothiazide	15.8	nitroglycerin nitrate	14.8

*Amlodipine 13.26% in those aged 75 to 84.

Forty-seven point two percent of patients aged 85 and older had at least one Beers-listed PIM, 63.3% at least one STOPP-listed PIM, 53.6% at least one START-listed PPO, and 59.4% at least one ACOVE-3-listed PPO, which was not significantly different to the group aged 75 to 84 (Table 3).

**Table 3:** **Prevalence of PIM and PPO according to the criteria**

**Criteria**	**85 years and over %**	**75 to 84 years old %**	**P**
Beers	47.28	53.76	P = 0.126
STOPP	63.36	60.47	P = 0.482
START	53.68	49.65	P = 0.342
ACOVE3	59.40	54.91	P = 0.289

In the oldest old patients, the most frequently found PIMs according to the Beers’ criteria was the use of short to intermediate acting benzodiazepines in patients with previous falls or syncope (10.7%) and the use of long-acting benzodiazepines independent of diagnoses or conditions (10.5%). The most commonly encountered STOPP criteria was the use of benzodiazepines in patients who are prone to falls (18.3%) and the use of long-term long-acting benzodiazepines (9.5%). Among the START criteria the most frequently identified PPO were ACE inhibitors in patients with heart failure (12.8%) and oral anticoagulation in the presence of chronic atrial fibrillation (12.8%). The most commonly identified PPO using the ACOVE criteria were calcium and vitamin D supplements in patients with osteoporosis (13.3%) and ACE inhibitors or angiotensin-receptor blockers in patients with hypertension and comorbid vascular diseases (10.8%).

With regard to PIM, the main difference according to the STOPP criteria was a higher prescription of benzodiazepines for patients with a history of falls in the group of the oldest old (18.4% versus 13.1% in those aged 75 to 84, p = 0.090), although the difference was not statistically significant. Regarding PPO, the main differences were a lower prescription of vitamin D and calcium supplements in patients with known osteoporosis in the group of the oldest old according to both the START (5.6% versus 11.3%, p = 0.013) and the ACOVE-3 criteria (5.7% versus 13.3%, p = 0.002), and a lower prescription of β-blocking agents in patients with arterial hypertension and ischemic heart disease according to the ACOVE-3 criteria (4.3% versus 10.6%, p = 0.002) (Table 4).

**Table 4:** **Main potentially inappropriate medicines (PIM) and potentially prescribing omissions (PPO) in patients aged 85 years and over compared with those aged 75 to 84 years**

**Disease or condition**	**Drug**	**85 years and over %**	**75 – 84 years %**	**p**
**Beers list PIM**				
Syncope or falls	Short- to intermediate-acting benzodiazepine and tricyclic antidepressants	10.7	9.8	0.712
Independent diagnosis	Long-acting benzodiazepines	10.5	12.7	0.268
**STOPP list PIM**				
Drugs that adversely affect those prone to falls	Benzodiazepines	18.4	13.2	0.090
Central nervous system and psychotropic drugs	Long-term (i.e. >1 month), long-acting benzodiazepines	9.5	11.7	0.687
Cardiovascular system	Aspirin at dose >150 mg day	8.6	4.9	0.106
Cardiovascular system	Aspirin with no history of coronary, cerebral or peripheral arterial symptoms or occlusive arterial event	7.8	7.5	0.909
**START list PPO**				
Cardiovascular system	ACE inhibitor with chronic heart failure	12.8	13.5	0.750
Cardiovascular system	Warfarin in the presence of chronic atrial fibrillation	12.8	10.3	0.343
Musculoskeletal system	Calcium and vitamin D supplement in patients with known osteoporosis	11.3	5.6	0.013
Endocrine system	Antiplatelet therapy in diabetes mellitus if one or more coexisting major cardiovascular risk factor present	8.8	10.3	0.530
**ACOVE 3 list PPO**				
Osteoporosis	IF a VE has osteoporosis, THEN he or she should be prescribed calcium and vitamin D supplements	13.3	5.7	0.002
Hypertension	IF a VE with HTN has a history of HF, left ventricular hypertrophy, IHD, chronic kidney disease, or cardiovascular accident, THEN he or she should be treated with an ACE inhibitor or ARB	12.6	9.9	0.289
Stroke and atrial fibrillation	IF a VE has chronic atrial fibrillation and is at medium to high risk for stroke, THEN anticoagulation should be offered.	10.7	8.1	0.242
Hypertension	IF a VE with HTN has IHD, THEN treatment with a beta-blocker should be recommended or documentation of why it should not be provided.	10.6	4.3	0.002
Osteoporosis	IF a female VE has osteoporosis, THEN she should be treated with bisphosphonates, raloxifene, calcitonin, hormone replacement therapy, or teriparatide	10.5	7.4	0.163

The results of the multivariate regression analysis are shown in Table 5. Only the statistically significant risk factors are presented. In the oldest old, a prescription of ten or more medicines was the most important independent factor associated with an increased risk of at least one Beers-listed PIM (OR 5.7, 95% CI 1.8-17.9). A prescription of ten or more medicines (OR 13.4, 95% CI 4.0-44.0) and a severe dependence in basic activities of daily living (OR 5.0, 1.1-22.1) were the independent factors associated with an increased risk of at least one STOPP-listed PIM. Instead, multimorbidity was associated with a reduced risk of at least one STOPP-listed PIM (OR 0.5, 0.2-0.9). In comparison to patients aged 75 to 84, female gender was not associated with an increased risk of PIM in the oldest old. In the oldest old, the independent factors associated with an increased risk of at least one START-listed PPO were a non-community dwelling (OR 2.3, 95% CI 1.0-5.0), and multimorbidity (OR 1.8, 1.0-3.1). The only factor associated with an increased risk of at least one ACOVE-3-listed PPO was the presence of at least one STOPP-listed PIM (OR 2.4, 1.4-4.3). These factors were also associated with PPO in the group of patients aged 75 to 84 with the exception of a non-community dwelling.

**Table 5:** **Results of the multivariate regression analysis**

**85 years old and over**			**75 to 84 years old**		
**Associated factor**	**OR (95% CI)**	**p**	**Associated factor**	**OR (95% CI)**	**p**
**Beers**			**Beers**		
Number of medicines			Number of medicines		
● 10 or more	5.7 (1.8-17.9)	0.003	● 5 - 9	6.3 (1.1-34.4)	0.035
			● 10 or more	11.0 (2.0- 59.7)	0.006
			Female gender	1.9 (1.1-3.1)	0.014
			START-listed PPO	1.8 (1.0-2.9)	0.027
**STOPP**			**STOPP**		
Number of medicies			Number of medicines		
● 5 - 9	5.7 (1.8-17.8)	0.003	● 10 or more	5.1 (1.5-16.8)	0.007
● 10 or more	13.4 (4.0-44.0)	<0.001	ACOVE-3-listed PPO	2.2 (1.2-3.6)	0.004
Severe dependence in					
ADL	5.0 (1.1-22.1)	0.031			
Multimorbidity	0.5 (0.2-0.9)	0.045			
**START**			**START**		
Non-community dwelling	2.3 (1.0-5.0)	0.030	Multimorbidity	1.9 (1.0-3.2)	0.027
Multimorbidity	1.8 (1.0-3.1)	0.040			
**ACOVE-3**			**ACOVE-3**		
STOPP-listed PIM	2.5 (1.4-4.3)	0.001	STOPP-listed PIM	2.2 (1.2-3.8)	0.004

Only the statistically significant risk factors associated to PIM and PPO tools are presented.

## Discussion

This study shows that in patients 85 years and older, polypharmacy and prevalence of inappropriate prescribing, both for PIM and PPO, were as high as those in the younger elderly. The assessed population was very elderly and with significant frailty, multimorbidity and dependence in ADL as has already been described in other studies [3,13,20].

In our study, the most frequently prescribed medicine for patients aged 85 and over was omeprazole, but without a significant difference in comparison with those aged 75 to 84. Among the most frequently prescribed medicines, the consumption of paracetamol, aspirin, amlodipine and lorazepam was higher and that of acenocoumarol and simvastatin lower in the very elderly in comparison to those aged 75 to 84. A lower use of anticoagulation therapy in the oldest old population with anticoagulation criteria is one of the most frequent causes of underprescribing in the elderly [15,21]. However, in our study no statistically significant differences were found between both age groups using the START and ACOVE-3 criteria.

It is noteworthy that for patients aged 85 and over, compared to the younger group, the main cause of PIM, both for the Beers and the STOPP criteria, was a higher use of benzodiazepines, especially in people with a history of falls detected using the STOPP criteria. The relationship between benzodiazepine use and an increased risk of falls is well known [22], as is the use of benzodiazepines being the leading cause of PIM [9,20], although most of these studies did not focus on the very elderly. Recently, some authors have looked at reducing prescribing benzodiazepines [23], and in some studies, benzodiazepines’ cessation has been associated with a reduction in falls [24]. However, these studies did not focus on the very elderly.

Regarding PPO, the main differences in comparison to patients aged 75 to 84, were a greater omission of calcium and vitamin D supplements in the oldest old with osteoporosis according to the START and the ACOVE-3 criteria, and a greater omission of β-blockers in the oldest old with arterial hypertension and ischemic heart disease using the ACOVE-3 criteria. A high prevalence of vitamin D deficiency in patients aged 85 years and over [25], and the association between hypovitaminosis D and various health problems in the elderly have been described [26]. Moreover, omission of calcium and vitamin D supplements in the elderly with osteoporosis has been the leading cause of PPO in different studies [21,27]. This is relevant, taking into account the benefit of calcium and vitamin D supplements in preventing bone loss and fractures in elderly people with osteoporosis [28,29], although once again, these studies were not performed on the very elderly. The higher omission of β-blockers in the oldest old with hypertension and ischemic heart disease may be due to more frequent use of other antihypertensives in this frail group.

The main factor associated with PIM in both age groups was polypharmacy, especially when 10 or more medicines were taken. The strong association between polypharmacy and PIMs [21,30] and also the higher prevalence of PIM when STOPP criteria was used in comparison to the Beers criteria have already been reported on in other studies [9,21]. The association between a severe dependency in ADL and PIM found in our study can be explained, at least in part, by the high prevalence of dementia in this population.

Regarding PPO in patients aged 85 years or older, the relevant findings in our study were the higher ACOVE-3 PPO criteria prevalence, and the association between a nursing home origin and multimorbidity with the START criteria. That is, patients from a nursing home and those with multimorbidity had a higher risk of underprescription. The lack of association between polypharmacy and PPO is well known [30], as is the scarce scientific evidence available regarding risk factors associated with underprescribing. The association with multimorbidity has also been described in other studies [21], and other described factors have been advanced age [19,21], and female gender [19]. The high proportion of PIM and PPO in the elderly institutionalized in nursing homes is known about [31]. Moreover, the implementation of the STOPP/START criteria has been associated with a reduction in the number of drugs and falls in this population [32]. The association between underprescribing and a nursing home origin found in our study is curious. In contrast, a low START-listed PPO prevalence in patients with dementia has been described [21]. The authors of this study argued that patients with dementia are probably more often institutionalized in nursing home facilities with more organized pharmaceutical care. More studies are needed to clarify the association between underprescribing and a nursing home origin.

Something new that we have found is a link between PIM and PPO in a very elderly population with polypharmacy. This may be explained because a large percentage of patients in our study had PIM and PPO at the same time [15]. However, this is a surprising finding because risk factors associated with PIM and PPO did not match in several other studies [15,18,19,21]. More information on risk factors simultaneously associated with PIM and PPO in very elderly patients with polypharmacy is needed. Moreover, the association between multimorbidity and PPO has been previously reported on in the elderly, but the inverse association with PIM is more striking. It seems that in the very elderly with multimorbidity, physicians avoid medicines included in the PIM criteria with a low level of scientific evidence on their efficacy and/or with better alternatives.

In the elderly patients with multimorbidity, and especially in the oldest old, the usefulness of underprescribing tools is controversial. A high prevalence of medicine omission is often described when they are systematically used in these patients [19,21], although, as discussed above, the available evidence on treatments of chronic diseases in these patients are low or insufficient [4].

Our study has several strengths. Firstly, it was carried out on a large group of very elderly people and is a topic that has infrequently been investigated. Secondly, a rigorous methodology in both the geriatric and the pharmacological assessment of patients was applied, and thirdly it was a multicentric study involving seven hospitals lasting a year.

This study also has some limitations. Firstly, the Beers’ criteria version 2003 was used, and currently there is a new one [33] which only appeared once the study was initiated. A new version of the STOPP/START criteria has also been published very recently, although the indicators identified in our study as the most prevalent in the oldest old remain unchanged in the new version [34]. Secondly, only patients admitted to medical units in hospitals were included and they are not representative of the very elderly community dwelling patients. Finally, the consequences of inappropriate prescribing were not analysed.

## Conclusions

In our study, a high prevalence of polypharmacy, PIM and PPO in patients aged 85 years and over has been described. In addition, regarding PIM, a higher prevalence when STOPP criteria were use in comparison to the Beers criteria, use of benzodiazepines as the primary cause for PIM, especially in people prone to falls, and a strong association with polypharmacy were found. Regarding PPO, omission of calcium plus vitamin D supplements in patients with osteoporosis as the primary cause for PPO, and a paucity of factors associated with PPO were reported on in this population. This multicentric study was carried out on a large cohort of the very elderly, an infrequently investigated group, and a comprehensive geriatric and pharmacological assessment of patients was applied. To improve the available information on the risk factors associated with PIM and especially with PPO, to analyze its consequences, to refine and adjust the PIM and PPO criteria, and finally, to carry out interventions to improve prescribing in this very elderly population, more research is needed.

### PIPOPS investigators

Coordinator group: Antonio San-José (main investigator), Antònia Agustí, Xavier Vidal, Cristina Aguilera, Elena Ballarín, Eulàlia Pérez, Xavier Barroso.

Hospital Universitari Vall D’hebron (Barcelona): José Barbé, Carmen Pérez Bocanegra, Ainhoa Toscano, Carme Pal, Teresa Teixidor.

Hospital San Juan de Dios de Aljarafe (Sevilla): Antonio Fernández-Moyano, Mercedes Gómez Hernández, Rafael de la Rosa Morales, María Nicolás Benticuaga Martínez.

Hospital Clínic (Barcelona): Alfonso López-Soto, Xavier Bosch, Mª José Palau, Joana Rovira, Margarita Navarro.

Hospital Universitari de Bellvitge (Hospitalet de Llobregat, Barcelona): Francesc Formiga, David Chivite, Beatriz Rosón, Antonio Vallano, Carme Cabot.

Hospital Juan Ramón Jiménez (Huelva): Juana García, Isabel Ballesteros.

Hospital Santa Creu i Sant Pau (Barcelona): Olga H. Torres, Domingo Ruiz, Miquel Turbau, Paola Ponte, Gabriel Ortiz.

Hospital Universitario Virgen Del Rocío (Sevilla): Nieves Ramírez-Duque, Paula-Carlota Rivas Cobas, Paloma Gil.

## Additional file

Additional file 1:
**Pharmacological anamnesis.** (If used, cited authors as Fundació Institut Català de Farmacologia. Barcelona. Spain.

## Abbreviations

ACE inhibitors: Angiotensin convertor enzyme inhibitors
ACOVE3: Assessing Care Of Vulnerable Elders 3
ADL: Activities of daily living
IP: Inappropriate prescription
PIM: Potentially inappropriate medicines
PPO: Potentially prescribing omissions
START: Screening tool to alert doctors to the right treatment
STOPP: Screening tool of older persons’ potentially inappropriate prescriptions
