# Supplementary material for: Inappropriate prescribing to the oldest old patients admitted to hospital: prevalence, most frequently used medicines, and associated factors
Source: BMC Geriatr. 2015 Apr 9;15:42. doi: 10.1186/s12877-015-0038-8 (PMC4403827; doi:10.1186/s12877-015-0038-8)
Supplement: Additional file 1: — Pharmacological anamnesis. (If used, cited authors as Fundació Institut Català de Farmacologia. Barcelona. Spain. [file 12877_2015_38_MOESM1_ESM.docx]

Additional file 1. Pharmacological anamnesis. (If used, cited authors as Fundació Institut Català de Farmacologia. Barcelona. Spain.

| **Pharmacological anamnesis** |
| --- |
| **The following questions refer to the use of medicines during the month prior to hospital admission.**  **1.- Open question to the patient: What medicines have you taken during the last month?**  **2.- Questions by indications: During this period have you taken something for…?**  **3.- Questions about clinical problems from the medical record: During this period have you taken anything for this problem/disease?**  **4.- Have you taken anything else during the last month?**   \| **Indications** \| \| \| --- \| --- \| \|  \| Headache/ migraine \| \|  \| Toothache/mouth \| \|  \| Osteoarthritis / knee pain, back, neck... \| \|  \| Arthritis / uric acid \| \|  \| Pain for blows / falls \| \|  \| Dyspepsia / abdominal pain / indigestion / ulcer \| \|  \| Constipation \| \|  \| Fatigue / malaise / apathy \| \|  \| other pains \| \|  \| Cold / cough / sore throat \| \|  \| Fever \| \|  \| Antibiotics / Infection \| \|  \| Asthma / breathing problems \| \|  \| Heart disease \| \|  \| Circulation problems / varices \| \|  \| Cholesterol \| \|  \| Hypertension \| \|  \| Diabetes \| \|  \| Nerves / insomnia / depression \| \|  \| Vitamines / To slim \| \|  \| Herbs, herbal medicine \| \|  \| Master formulas \| \|  \| Ear problems / eye drops \| \|  \| Prostate Problems \| \|  \| Thyroid problems \| \|  \| Others \| |
